# Characterization of leucine aminopeptidase (LAP) activity in sweet pepper fruits during ripening and its inhibition by nitration and reducing events

**DOI:** 10.1007/s00299-024-03179-x

**Published:** 2024-03-11

**Authors:** María A. Muñoz-Vargas, Jorge Taboada, Salvador González-Gordo, José M. Palma, Francisco J. Corpas

**Affiliations:** Department of Stress, Development and Signaling in Plants, Group of Antioxidants, Free Radicals and Nitric Oxide in Biotechnology, Food and Agriculture, Estación Experimental del Zaidín Spanish National Research Council, CSIC, C/Profesor Albareda, 1, 18008 Granada, Spain

**Keywords:** Aminopeptidase, Cyanide, Fruit ripening, Glutathione, Intron/exon, Nitration, Nitric oxide, Pepper

## Abstract

**Key message:**

**Pepper fruits contain two leucine aminopeptidase (*LAP*) genes which are differentially modulated during ripening and by nitric oxide. The LAP activity increases during ripening but is negatively modulated by nitration**.

**Abstract:**

Leucine aminopeptidase (LAP) is an essential metalloenzyme that cleaves N-terminal leucine residues from proteins but also metabolizes dipeptides and tripeptides. LAPs play a fundamental role in cell protein turnover and participate in physiological processes such as defense mechanisms against biotic and abiotic stresses, but little is known about their involvement in fruit physiology. This study aims to identify and characterize genes encoding LAP and evaluate their role during the ripening of pepper (*Capsicum annuum* L.) fruits and under a nitric oxide (NO)-enriched environment. Using a data-mining approach of the pepper plant genome and fruit transcriptome (RNA-seq), two *LAP* genes, designated *CaLAP1* and *CaLAP2*, were identified. The time course expression analysis of these genes during different fruit ripening stages showed that whereas *CaLAP1* decreased, *CaLAP2* was upregulated. However, under an exogenous NO treatment of fruits, both genes were downregulated. On the contrary, it was shown that during fruit ripening LAP activity increased by 81%. An in vitro assay of the LAP activity in the presence of different modulating compounds including peroxynitrite (ONOO^−^), NO donors (*S*-nitrosoglutathione and nitrosocyteine), reducing agents such as reduced glutathione (GSH), l-cysteine (l-Cys), and cyanide triggered a differential response. Thus, peroxynitrite and reducing compounds provoked around 50% inhibition of the LAP activity in green immature fruits, whereas cyanide upregulated it 1.5 folds. To our knowledge, this is the first characterization of LAP in pepper fruits as well as of its regulation by diverse modulating compounds. Based on the capacity of LAP to metabolize dipeptides and tripeptides, it could be hypothesized that the LAP might be involved in the GSH recycling during the ripening process.

**Supplementary Information:**

The online version contains supplementary material available at 10.1007/s00299-024-03179-x.

## Introduction

Aminopeptidases (APs) are conserved enzymes that range from bacteria to higher organisms and catalyze the cleavage of an amino acid at the N-terminus of a peptide or protein. They play an essential role in cell protein turnover but also in physiological processes such as defense mechanisms against biotic and abiotic stresses (Matsui et al. 2006; Waditee-Sirisattha et al. 2011). In higher plants, research on APs has been focused on processes such as seed germination or in response to different stresses (Jaouani et al. 2018; Kania et al. 2021). APs also participate in the redistribution of amino acids from storing sink tissues during germination, although little is known about the role of AP in this process (Chao et al. 1999), as well as on their subcellular localization (Corpas et al. 1993; Narváez-Vásquez et al. 2008). Other APs participate in organelle amino acid recovery since they can release various N-terminal amino acids such as alanine, leucine, or aspartic from short peptides in both mitochondria and chloroplasts (Teixeira and Glaser 2013; Park et al. 2017). More recently, it has been characterized in *Arabidopsis thaliana* a mitochondrial AP that can release proline which is then needed for the tolerance to abiotic stresses as well as leaf senescence and the development of male gametophytes (Zdunek-Zastocka et al. 2017; Kmiec et al. 2018; Ghifari et al. 2020, 2022). Leucine aminopeptidase (LAP) is a metalloenzyme that cleaves N-terminal residues of proteins, more specifically Leu, Met, or Arg, but they can also hydrolyze dipeptides such as Cys–Gly into free amino acids (Gu and Walling 2000; Kumar et al. 2015). LAP belongs to the family of M1 or M17 peptidases which are characterized by having Zn^2+^ in their structure. The binding to Zn^2+^ involves two histidine residues that are present in the HEXXH consensus sequence, a third residue of variable nature, and a water molecule. Plant LAPs can be classified as acidic LAP-As or neutral LAP-Ns according to their pI. LAP-As and LAP-Ns have different biochemical properties and respond differently to developmental and environmental signals. LAP-As seem to appear only in the Solanaceae, and they are induced by biotic and abiotic stresses and accumulate in reproductive organs (Herbers et al. 1994; Chao et al. 1999, 2000; Scranton et al. 2012; Panpetch and Sirikantaramas 2021).

Nitric oxide (NO) is a gasotransmitter that is generated endogenously in plant cells and exerts a myriad of regulatory functions under physiological and stressful conditions (Leterrier et al. 2012; Kolbert et al. 2019; Parveen et al. 2023). NO has a family of derived molecules designated as reactive nitrogen species (RNS). Among these molecules, we can highlight peroxynitrite (ONOO^−^) and *S*-nitrosoglutathione (GSNO), which are the results of the interaction of NO with the superoxide radical (O_2_^•−^) and with reduced glutathione (GSH), respectively. However, one of the most controversial aspects of NO in higher plants is how it is enzymatically generated. At present, there are two main enzymatic sources including nitrate reductase (NR) which generates NO from nitrite and nitrate using NADH as an electron donor (Yamasaki and Sakihama 2000; Mohn et al. 2019). On the contrary, there is an l-arginine-dependent NO-like synthase that needs all the cofactors required for the animal NOSs including NADPH, FMN, FAD, tetrahydrobiopterin, calmodulin, and calcium (Corpas et al. 2022a).

Pepper (*Capsicum annuum* L.) fruits are framed within the Solanaceae family and have great agroeconomic relevance worldwide since they are broadly consumed either fresh or processed. For example, dry pepper powder is one of the most used spices in the preparation of cooking dishes for its aroma and flavor, but also in the preparation of sausages both for its antioxidant capacity that provides a high preservative capacity and for its taste. In previous studies, we have provided evidence of the relevance of the metabolism of reactive oxygen and nitrogen species (ROS and RNS, respectively) in the ripening of sweet pepper fruits and how the application of exogenous NO could modulate these processes at different levels (Rodríguez-Ruiz et al. 2017; González-Gordo et al. 2019). Thus, it has been shown that enzymes such as peroxidases, superoxide dismutases (SODs), NADPH oxidases, and ascorbate peroxidases (APXs), among others, underwent numerous changes at the transcriptomic, proteomic, and biochemical levels (Rodríguez-Ruiz et al. 2017; González-Gordo et al. 2022, 2023; Muñoz-Vargas et al. 2023a, b). The present study focuses on the identification and characterization of the LAP in sweet pepper fruits and its modulation during their ripening since, to our knowledge, there is no previous information about this metalloprotease in this plant species. Thus, two genes have been identified, *CaLAP1* and *CaLAP2*, and it has been evaluated how their expression is modulated during ripening and by the effect of exogenous application of NO. Likewise, at a biochemical level, it is shown that LAP activity increases during ripening and how certain molecules such as ONOO^−^, NO donors, cyanide (CN^−^), and reducing compounds such as GSH and l-cysteine (l-Cys) differentially modulate LAP activity.

## Plant materials and methods

### Plant material and exogenous nitric oxide (NO) gas treatment of fruits

California-type sweet pepper (*C. annuum* L., cv. Melchor) fruits were harvested between January and February (2021) from plants grown in plastic-covered greenhouses (Syngenta Seeds, Ltd., Roquetas de Mar/El Ejido, Almería, Spain). As previously described (González-Gordo et al. 2019), fruits were selected and harvested without any external apparent injury at three developmental stages: green immature (G), breaking point (BP1), and red ripe (R). For the analysis of the exogenous NO gas treatment, two additional groups were established: fruits treated with 5 ppm NO for 1 h (BP2 + NO) and fruits not treated with NO (BP2 − NO). After 3 days at room temperature, all fruits were chopped into small cubes (5 mm/edge), frozen under liquid nitrogen, and stored at − 80 °C until use. Supplementary Figure S1 shows the experimental design followed in this study with the representative phenotypes of sweet pepper fruits at different ripening stages and subjected to NO treatment (González-Gordo et al. 2022).

### Library preparation, RNA-sequencing, and transcriptome analyses

Pepper fruit libraries were prepared using an optimized Illumina protocol and were sequenced on an Illumina NextSeq550 platform using 75 bp paired-end reads as previously described (González-Gordo et al. 2019). Briefly, reads were pre-checked to eliminate low-quality sequences and with these clean reads, the de novo transcriptome assembly was accomplished. Bowtie2 tool was used to realign the reads, whereas Samtools was used to quantify the known transcripts. Differential expression analyses were carried out using TransFlow (Seoane et al. 2018) and DEgenes-Hunter (Gayte et al. 2017), which use diverse algorithms with their own statistical tests to validate the whole experiment (González-Gordo et al. 2019). Sequence Read Archive (SRA) data are available at the following link: https://www.ncbi.nlm.nih.gov/sra/PRJNA668052 (accessed on May 28, 2023).

### Identification of *LAP* genes in pepper, analysis of the *CaLAP* intron–exon structure and *cis*-regulatory elements, and chromosomal location

To identify the *LAP*-encoding genes, pepper proteome was downloaded from the NCBI database (Assembly UCD10Xv1.1; BioProject PRJNA814299). Furthermore, the amino acid sequences from the LAPs described in *Arabidopsis*, rice, and pea were downloaded from the UnirProtKB database (see Supplementary Table S1 for the Protein ID of these LAPs) (accessed on February 10, 2023). These sequences were used as a query to search for LAPs in the complete pepper proteome using the BLASTP tool. Location coordinates of the identified *CaLAP* genes in the pepper genome were obtained from the NCBI database.

The promoter sequences of the *CaLAPs* were obtained from the NCBI Nucleotide database (https://www.ncbi.nlm.nih.gov/nucleotide/) considering 2000 bp upstream from the transcription starting point of each gene. These sequences were searched for possible *cis*-acting regulatory elements using the PantCARE tool (Lescot et al. 2002). These results were manually processed and visualized using the “Basic Biosequence View” function of TBtools v1.108 software (Chen et al. 2020). The chromosomal location of the identified *CaLAPs* was withdrawn from the NCBI database and the corresponding genetic map was constructed using the MG2C_v2.1 tool (Chao et al. 2021).

### Evolutionary analysis by maximum likelihood method and conserved motif analyses of CaLAP protein sequences

The identified CaLAP protein sequences in pepper as well as those from tomato (*Solanum lycopersicum* L.), potato (*Solanum tuberosum*), grapes (*Vitis vinifera* L.), pea (*Pisum sativum* L.), durian (*Durio zibethinus*), tobacco (*Nicotiana tabacum*), olive (*Olea europeae*), rice (*Oryza sativa* L.), and *Arabidopsis thaliana* were used to construct a phylogenetic tree (Supplementary Table S1). The evolutionary history was inferred using the maximum likelihood method and JTT matrix-based model (Jones et al. 1992). The bootstrap consensus tree inferred from 1000 replicates (Felsenstein 1985) was taken to represent the evolutionary history of the taxa analyzed. Branches corresponding to partitions reproduced in less than 50% of bootstrap replicates were collapsed. Initial tree(s) for the heuristic search were obtained automatically by applying neighbor-join and BioNJ algorithms to a matrix of pairwise distances estimated using the JTT model and then selecting the topology with superior log-likelihood value. This analysis involved 26 amino acid sequences. There were a total of 682 positions in the final dataset. Evolutionary analyses were conducted in MEGA11 (Tamura et al. 2021).

The alignment of LAPs was performed using the CLUSTALW method. Then, the aligned sequences were subjected to MEGA11 v0.13 to perform an unrooted maximum likelihood phylogenetic tree with default parameters (Tamura et al. 2021). Finally, the resulting phylogenetic tree was modified using the online tool iTOL (Letunic and Bork 2006). The protein localization based on their amino acid sequences was predicted using WoLF PSORT (Horton et al. 2007). Computational prediction of tyrosine nitration sites in CaLAP1 was done with the GPOS-YNO2 software (Liu et al. 2011).

### Protein modeling of CaLAPs

The native structure of the CaLAPs was predicted by the artificial intelligence (AI) software AlphaFold (Jumper et al. 2021). The residues involved in substrate binding, catalytic function, and the potential site of nitration were visualized using RasTop software (https://www.geneinfinity.org/rastop/) (accessed on July 15, 2023).

### Fruit extracts and leucine aminopeptidase (LAP; EC 3.4.11.1) enzyme activity

Fruit samples stored at − 80 °C were homogenized in liquid nitrogen using an IKA A11 Extraction Mill. The pulverized plant material was weighed and then added 50 mM Tris–HCl buffer, pH 7.5, containing 0.1 mM EDTA, 1 mM MgCl_2_, 10% (v/v) glycerol, and 0.10% Triton X-100 (v/v). The ratio of plant material:buffer used was 1:1.

For spectrophotometric LAP activity assay, l-leucine-*p*-nitroanilide (Leu-*p*-NA) was used as a substrate which, by the action of the LAP activity, generates l-Leu plus *p*-nitroaniline whose absorbance is measured at 410 nm. Briefly, the reaction mixture contained 50 mM potassium phosphate buffer pH 7.5, 10 mM β-mercaptoethanol, and 1 mM Leu-*p*-NA plus plant samples and were incubated at 39 °C for 30 min. The reaction was stopped with 30% (v/v) acetic acid and centrifuged at 10,000 g for 10 min, and then the supernatant was measured at 410 nm (Corpas et al. 1993). One unit of LAP activity is defined as a change in one unit of absorbance at 39 °C which corresponds to the production of 1 nmol of *p*-nitroaniline per min, using an extinction coefficient ε_410_ for the *p*-nitroaniline of 10^4^ M^−1^ cm^−1^ (Tuppy et al. 1962).

### Statistical analysis

Data of enzymatic activity were presented as the mean ± SEM of three independent biological replicas. For comparisons of the LAP activity among treatments compared to control, the Student’s *t* test was used. Values of *P* < 0.05 were considered statistically significant.

### In vitro treatment with NO donors, ONOO^−^, reducing agents, and cyanide

For the in vitro assays, samples from green pepper fruits were pre-incubated with different potential modulators, including SIN-1 (hydrochloride, 3-Morpholinosydnonimine); a ONOO^−^ donor; nitrating compounds––GSNO (nitrosoglutathione) and CysNO (nitrosocysteine) as NO donors; l-Cys and GSH––as reducing compounds as well as internal control for the NO donors; and potassium cyanide (KCN). In all cases, the solutions were freshly prepared before use at a concentration of 5 mM, and the treatments were done at 25 °C for 1 h in the dark, except the treatment with SIN-1, which was done at 37 °C for 1 h.

The NO release from 5 mM of GSNO and CysNO was corroborated by electron paramagnetic resonance spectroscopy (EPR) using the spin trap Fe(MGD)_2_. The spin trap was prepared by mixing *N*-methyl-d-glucamine dithiocarbamate (MGD) and FeSO_4_ stock solutions to give a final concentration in the reaction mixture of 10 mM and 1 mM, respectively (Corpas et al. 2004). The EPR spectrum was obtained using a Bruker Magnettech ESR5000 spectrometer.

## Results

To identify the *LAP* genes in pepper, the databases of the available LAP sequences from several plant species were checked. Then, the *C. annuum* L. genome was mined, and this allowed the identification of two *LAP* genes, designated *CaLAP1* and *CaLAP2*, according to their chromosomal distribution. The data mining in the transcriptome previously obtained from sweet pepper fruits (González-Gordo et al. 2019) showed that both genes were expressed in fruits. Table 1 displays some properties of these genes and their corresponding encoded LAP proteins including the number of amino acids (aa), molecular mass (kDa), and their putative subcellular localization, among other features. Thus, based on the pI, CaLAP1 can be categorized as neutral LAP-N, whereas cytosolic CaLAP2 would be an acidic LAP-A.

**Table 1:** Leucine aminopeptidase (*LAP*) genes identified in the pepper genome with chromosomal location and some of the properties related to the protein encoded for these genes, including the number of amino acids (aa), molecular mass (kDa), theoretical pI, and their subcellular localization

Gene name	Gene ID	Chr	Protein ID	Length (aa)	kDa	pI	Subcellular location
*CaLAP1*	107840870	9	XP_016540297.2	584	60,997	7.91	Plastid/mito
*CaLAP2*	107847521	11	XP_016547301.1	613	68,808	5.05	Cytosol

*Chr* chromosome

**Fig. 1:**
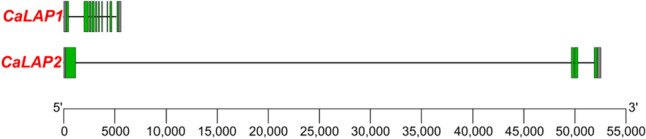
Genomic organization of the *CaLAP* genes. The gene structure is displayed with exons (green boxes) and introns (black lines). Untranslated regions are shown in gray boxes. Exon–intron regions are drawn at scale (colour figure online)

Figure 1 illustrates the genomic organization of the *CaLAP* genes. *CaLAP1* contained ten exons, whereas *CaLAP2* displayed four exons. Remarkably, the length of the introns was very different among both *CaLAPs*, with *CaLAP2* being the gene with a very long intron expanding above 50 kb.

**Fig. 2:**
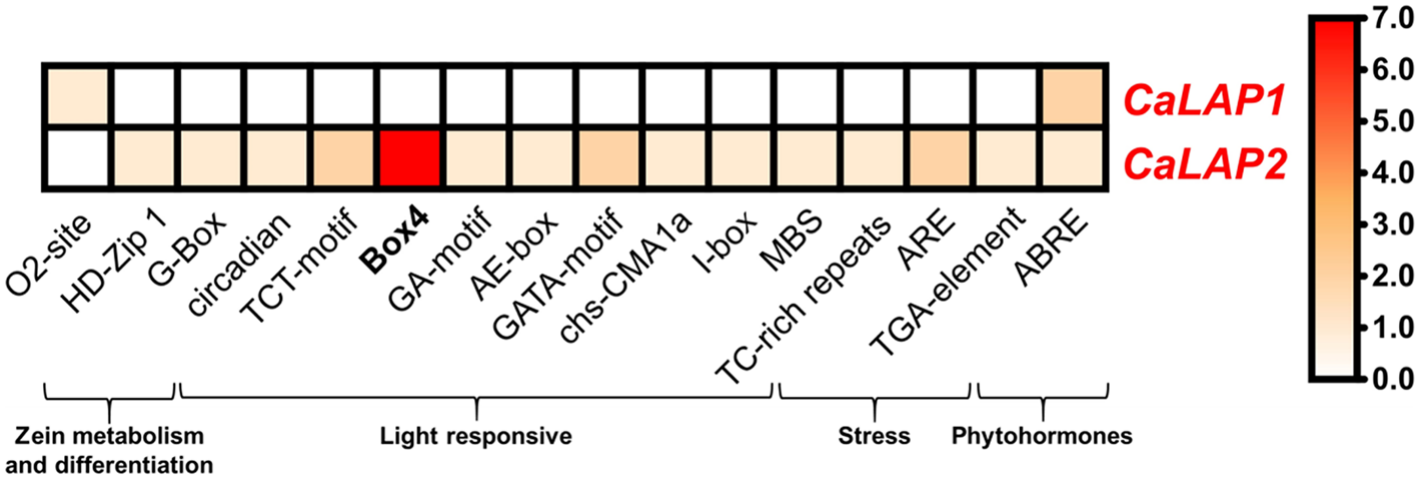
Heatmap of *cis*-regulatory elements corresponding to the 2000 bp upstream regions of both *CaLAP* genes. The *cis*-regulatory elements were assembled according to their functional implications including zein metabolism, light responsiveness, stress, and phytohormones. *Cis*-regulatory elements were identified in the PlantCARE database

Figure 2 depicts the heatmap analysis of the 16 identified *cis*-regulatory elements of both *CaLAP* genes that were grouped into four families. They include zein metabolism and differentiation, light responsiveness, stress, and phytohormones. The *cis*-regulatory element that exerts the most remarkable effects (reddish squares) was Box4 for *CaLAP2*. The detection of the O_2_-site in *CaLAP2* is significant, which corresponds to Opaque2, an endosperm-specific transcription factor (TF) belonging to the bZIP family which regulates the *22-kD α-zein* and *15-kD β-zein* genes by recognizing the O_2_ box (TCCACGT) in their promoters. Zein proteins are related to some of the most abundant cereal seed storage proteins (SSPs) (Zhang et al. 2015).

### Phylogenetic and protein sequence analysis of CaLAPs

**Fig. 3:**
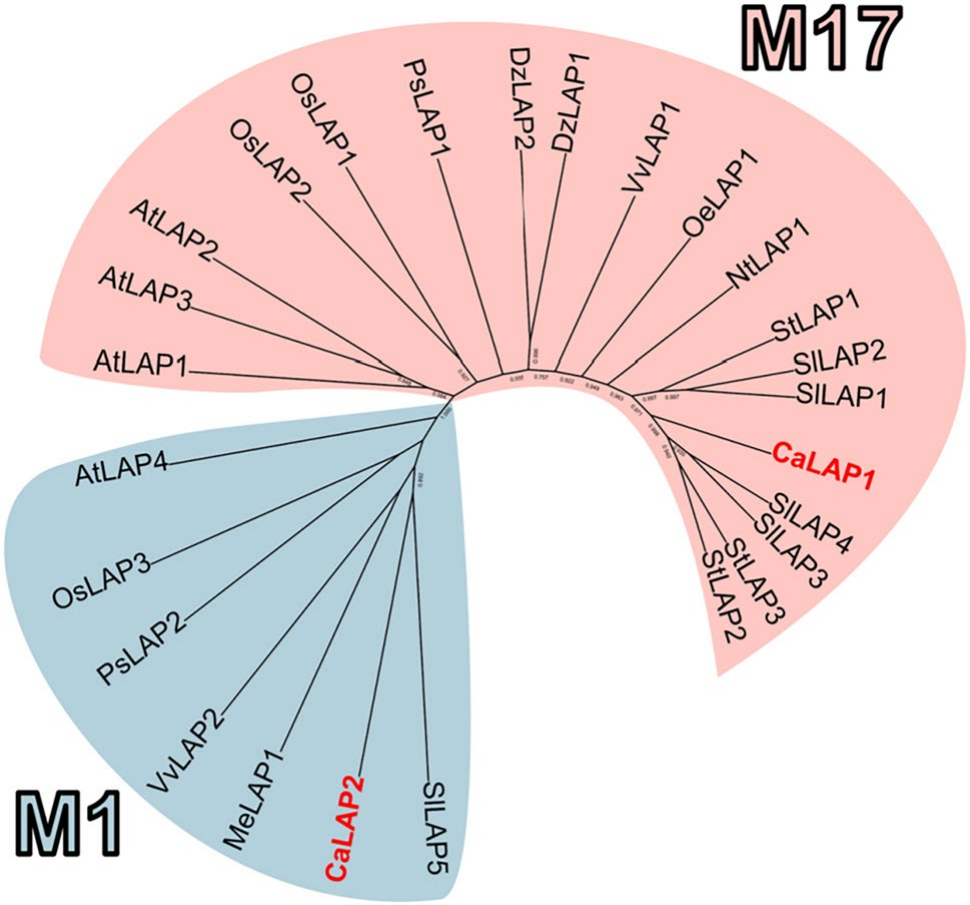
Phylogenetic tree of 15 LAPs from ten different plant species. Group M17 contains the CaLAP1, while group M1 contains the CaLAP2. The LAPs found specifically in the sweet pepper fruit are indicated in red. Species abbreviations: At, *Arabidopsis thaliana*; Ca, *Capsicum annuum*; Dz, *Durio zibethinus* Nt, *Nicotiana tabacum*; Oe, *Olea europea*; Os, *Oryza sativa*; Ps, *Pisum sativum*; Sl, *Solanum lycopersicum*; St, *Solanum tuberosum*; Vv, *Vitis vinifera* (colour figure online)

The comparative analysis of the CaLAP proteins with some LAPs from other plant species allowed us to draw a phylogenetic tree where two groups of LAPs corresponding to the M17 and M1 families previously mentioned were distinguished (Fig. 3). Supplemental Table 1 collects all the LAP sequences used with their corresponding protein ID.

**Fig. 4:**
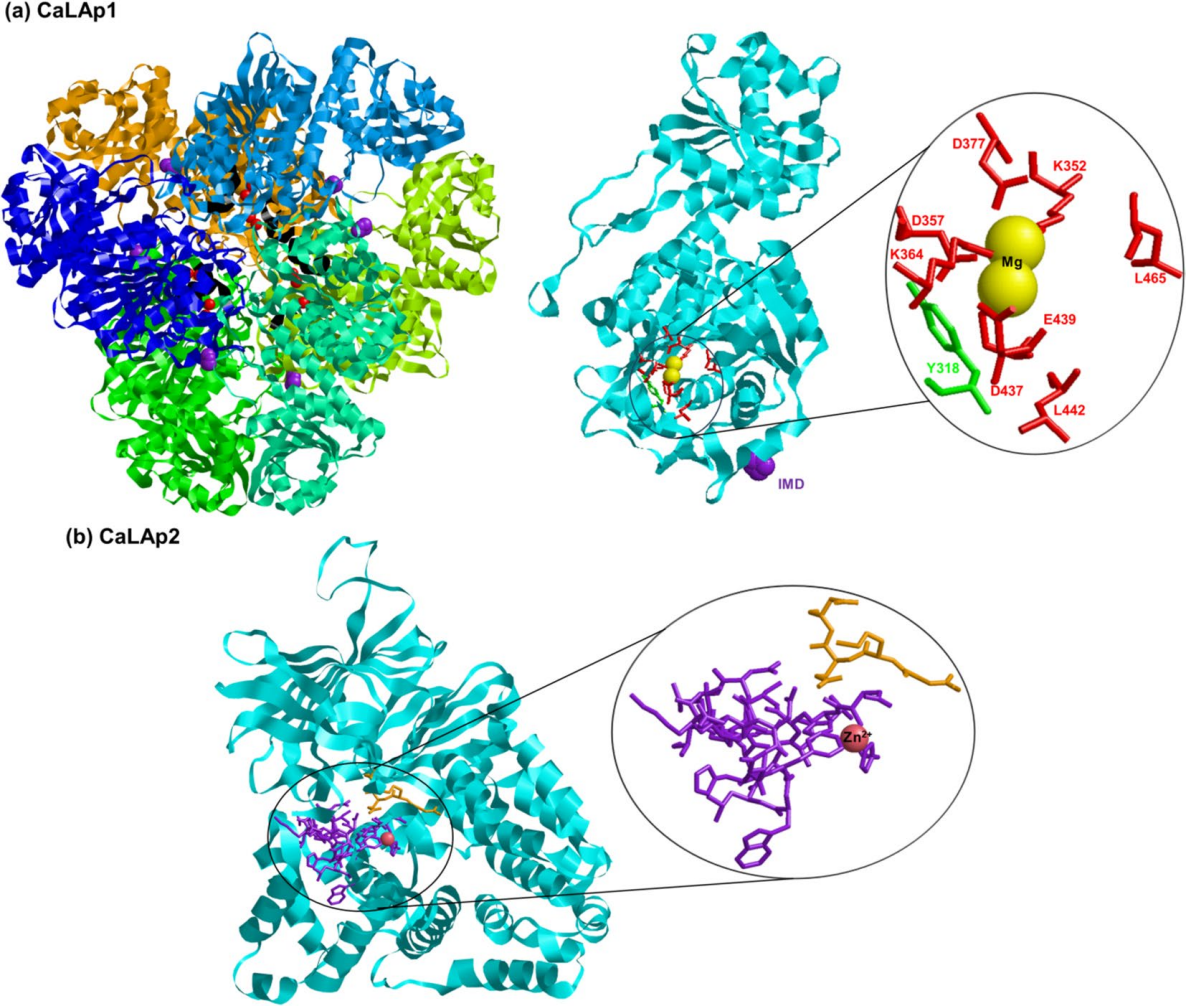
Molecular model of the plastid CaLAP1 and CaLAP2. **a** Model of the predicted homohexamer of CaLAP1 (left), monomer tertiary structure (center), and a detail of the eight highly conserved residues involved in substrate binding or the catalytic function (right) around Mg^2+^ which correspond to Lys350, Asp357, Lys364, Asp377, Asp437, Glu439, Arg441, and Leu465 (red). The Tyr318 (green) is labeled as a potential site of nitration. **b** Model of the CaLAP2. The Zn-binding site (residues 305–327) appears in purple and the region corresponding to the exopeptidase (residues 278–282) appears in orange (colour figure online)

When the protein sequences of plastidial CaLAP1 and cytosolic CaLAP2 were compared, it was found that the percentage of identity is very low (7%), with very long gaps possibly due to the great difference in the structure of both genes and probably affecting their functions. Therefore, as part of this analysis, we focused on the protein alignment of the plastidial CaLAP1 with LAP protein sequences from different species, where LAP proteins have been identified in their fruits. They include tomato, durian, and olive (Supplementary Fig. S2). Thus, eight highly conserved residues involved in substrate binding or catalytic function were identified corresponding to K350, D357, K364, D377, D437, E439, R441, and L465 in the CaLAP1 (labeled in red letters in Fig. 4a). Furthermore, five of these eight residues are conserved metal ion-coordinating residues (brown boxes). With this information, the predicted tertiary structure as a homohexamer of the plastid CaLAP1 was built (Fig. 4a) where the more relevant residues involved in the active site as well as the metal-binding locus were indicated. On the contrary, Fig. 4b illustrates the model of the CaLAP2 where the Zn^2+^-binding site (residues 305–327) is remarked in purple and the region corresponding to the exopeptidase (residues 278–282) in orange.

### Expression of *CaLAP* genes during fruit ripening and effect of NO treatment

**Fig. 5:**
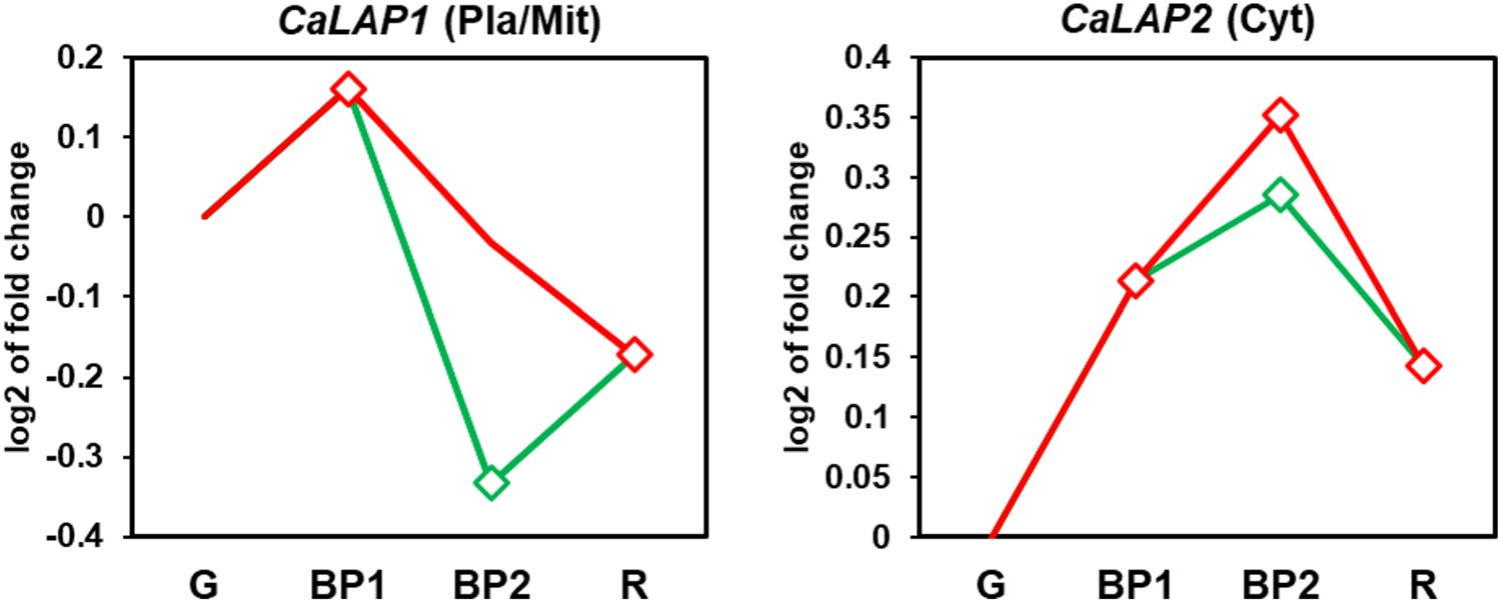
Time-course expression analysis of two *CaLAP* (*CaLAP1 and CaLAP2*) genes (RNA-Seq) under natural ripening conditions and after exogenous NO treatment. Samples of sweet pepper fruits at different ripening stages correspond to immature green (G), breaking point 1 (BP1), breaking point 2 after NO treatment (BP2 + NO, green line) and with no treatment (BP2 – NO, red line), and ripe red (R). Statistically significant changes in expression levels (*P* < 0.05) compared to green fruit (G) are indicated in diamonds (colour figure online)

Using the RNA-seq approach to sweet pepper fruits, the analysis of the two *CaLAP* genes was conducted at the different ripening stages and after the exposure of the fruits to exogenous NO gas. Supplementary Figure S2 shows representative pictures of the phenotype of pepper fruits during the experimental design which included green immature (G), breaking point (BP1), and red ripe (R) pepper fruits. Furthermore, two additional groups were established: fruits treated with 5 ppm NO for 1 h (BP2 + NO) and another untreated group (BP2 − NO) which corresponded to the control group against the NO-treated fruits. Figure 5 depicts the time-course analysis of the two *CaLAP* genes during ripening. Thus, whereas the *CaLAP1* expression was downregulated, the expression of *CaLAP2* was upregulated during this physiological process. However, under the exogenous NO treatment of the fruits, both genes were downregulated.

### Biochemical analysis of the LAP activity in fruits

**Fig. 6:**
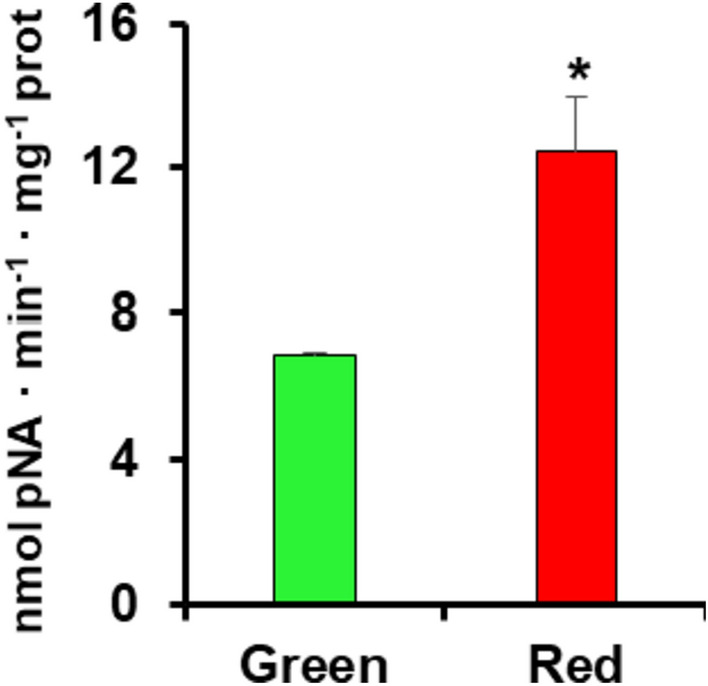
Leucine aminopeptidase (LAP) activity in green and red pepper fruits. Data are the mean ± SEM of at least three independent biological replicates. Asterisks indicate that differences between values were statistically significant at *P* < 0.05 (colour figure online)

As part of the biochemical characterization of the LAP enzymatic system, the total activity in green immature and red ripe pepper fruits was assayed. Figure 6 displays that LAP activity experimented an 80% increase in ripe fruits in comparison to green ones.

**Fig. 7:**
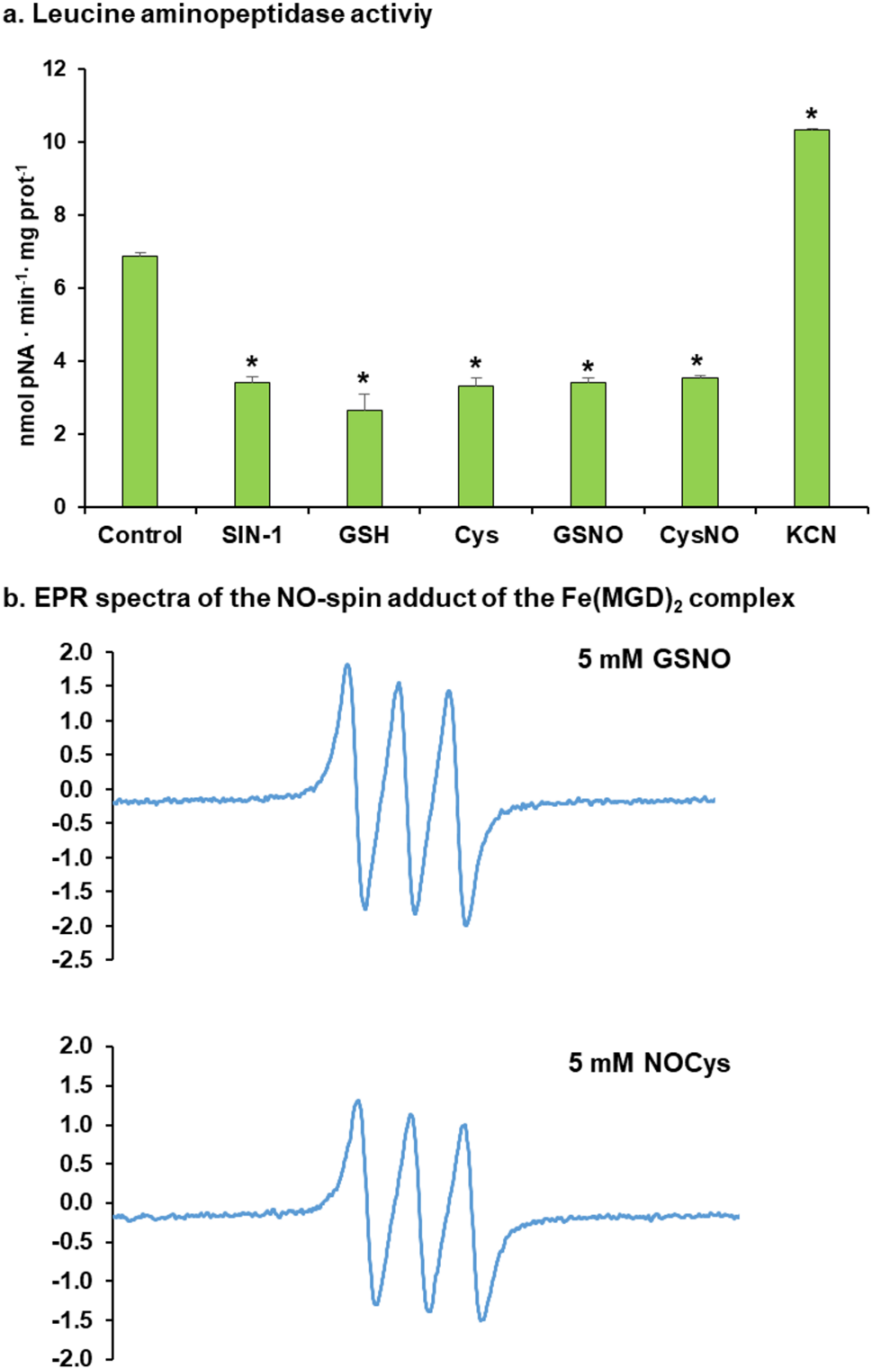
**a** Effect of different compounds on the LAP activity of green pepper fruits. SIN-1 (3-morpholinosydnonimine) is a peroxynitrate (ONOO^−^) donor and a nitrating compound. Nitrosoglutathione (GSNO) and nitrosocyteine (CysNO) are NO donors and nitrosating agents. Reduced glutathione (GSH) and l-cysteine (l-Cys) are reducing agents. Potassium cyanide (KCN) is an inhibitor of the mitochondrial electron transport chain. All treatments were done by pre-incubating the green pepper samples with these compounds (5 mM) at 25 °C for 1 h, except with SIN-1, which was pre-incubated at 37 °C for 1 h. Data are the mean ± SEM of at least three independent biological replicates. Asterisks indicate that differences between values were statistically significant at *P* < 0.05. **b** Representative EPR (electron paramagnetic resonance) spectra of the NO-spin adducts of the Fe(MGD)_2_ complex obtained from the NO release from 5 mM GSNO and 5 mM CysNO. The EPR parameters of the spectra were center field = 3378.95 G, sweep width = 200 G, gain = 40 dB, and sweep time = 30 s

To gain a deeper knowledge of the role of LAP in the physiology of pepper fruits, the regulation of LAP activity by different modulating compounds was also investigated by in vitro assays. Thus, samples from green pepper fruit samples were pre-incubated with a battery of reagents including the peroxynitrite (ONOO^−^) donor SIN-1, as a nitrating compound; the NO donors and nitrosating agents GSNO (*S*-nitrosoglutathione) and CysNO (nitrosocysteine); the reductants l-Cys and reduced GSH; and potassium cyanide (KCN), a compound which inhibits key enzymes involved in the mitochondrial electron transport chain (cytochrome c oxidase) and the ROS metabolism (CuZn-SOD), although it is also capable of mediating a post-translational modification (PTM) called *S*-cyanylation. Figure 7a shows that the LAP activity was inhibited between 50% and 60% by all compounds except KCN, which surprisingly increased it by about 50%. The effect of SIN-1 causing nitration of Tyr residues generally results in a loss of function as observed, thus suggesting that LAP underwent a nitration process. Using the GPS-YNO_2_ software (https://gps-yno2.software.informer.com/), among the ten tyrosines present in the CaLAP sequence, Tyr318 was identified with higher confidence as a nitration candidate. This tyrosine is close to the residues LysK364, AspD437, GluE439, and LysK364, which are part of the enzyme active center. Thus, Tyr318 nitration could justify the observed inhibition by peroxynitrite, although this must be corroborated by specific experiments using purified CaLAP1 and mass spectrometry analysis. In the case of the NO donors GSNO and CysNO, the activity was also reduced. It must be noticed that when these compounds release NO (Fig. 7b), GSH and Cys are also concomitantly released and, for this reason, these latter chemicals were also tested independently as internal controls. Based on the obtained data, it was observed that NO per se does not seem to exert any effect on the LAP activity of pepper fruits; however, both GSH and l-Cys do cause a significant inhibition.

## Discussion

The genus Capsicum includes approximately 25 species, of which there are five that have undergone a domestication process including *C. annuum*, *C. baccatum*, *C. chinense, C. frutescens*, and *C. pubescens*. Among them, *C. annuum* is the most agronomically important since it is extensively cultivated and consumed around the world (Nimmakayala et al. 2016; Antonio et al. 2018). Likewise, *C. annuum* has hundreds of varieties and, among other characteristics, two large groups can be made sweet and spicy according to the content of capsaicin (Vázquez-Espinosa et al. 2020). On the contrary, the ripening process of pepper fruits involves many changes at the phenotypic, transcriptomic, proteomic, and metabolomic levels, and its big genome size, in comparison to other Solanaceae, makes its analysis complex (Dyachenko et al. 2020; Razo-Mendivil et al. 2021; Esposito et al. 2022; Kovács et al. 2022; Del Giúdice et al. 2023). Recently, “omic” approaches have been carried out to study specific aspects of the ripening process (Chiaiese et al. 2019; Dubey et al. 2019; Zuo et al. 2019; Lopez-Ortiz et al. 2021; Rödiger et al. 2021; Momo et al. 2022; Villa-Rivera et al. 2022; Song et al. 2022; Liu et al. 2023; Wang et al. 2024) which are dependent on the variety, environmental growth conditions, fruit position, and ripening stage (Ribes-Moya et al. 2020; Jang et al. 2022; Lahbib et al. 2023; Guijarro-Real et al. 2023; Islam et al. 2023). Despite the increase in information on the ripening of pepper fruits, to our knowledge, there is no data on the LAP in this process.

The characterization of LAP has been achieved in different plant organs and species such as barley (*Hordeum vulgare* L.) (Kolehmainen and Mikola 1971; Oszywa et al. 2013), rice (Gupta and Pawar 1974), grapes (Pallavicini et al. 1981; Kang et al. 1999), pea (Elleman 1974; Corpas et al. 1993), oat (*Avena sativa*) (Casano et al. 1989), potato (Herbers et al. 1994), tomato (Gu et al. 1996; Gu and Walling 2000; Pautot et al. 2001; Tu et al. 2003), daylily (*Hemerocallis fulva* L.) (Mahagamasekera and Leung 2001), or *Arabidopsis* (Bartling and Weiler 1992; Polge et al. 2009; Waditee-Sirisattha et al. 2011). In the case of pepper (*C. annuum*), a recent proteomic study carried out on leaves affected by the aphid *Myzus persicase* detected an increase of 1.9 folds in the protein expression of a chloroplastic LAP (Florencio-Ortiz et al. 2021). However, to our knowledge, the presence of LAP in pepper fruits has not been studied so far. Therefore, the obtained data in this work provide new information on the gene, protein, and biochemical characterization of this protease in these non-climacteric fruits. Thus, the analysis of the pepper fruit transcriptome allowed the identification of two *LAP* genes and, based on the pI of the encoding proteins, the plastidial CaLAP1 could be categorized as a neutral LAP-N, whereas cytosolic CaLAP2 would be an acidic LAP-A. Previous studies in different plant species suggest that LAP-N is constitutively expressed in all plants, whereas LAP-A only appears in the Solanaceae, and its activity is induced under abiotic and biotic stresses (Gu et al. 1996; Gu and Walling 2000; Chao et al. 2000; Pautot et al. 2001). The phylogenetic analysis includes CaLAP1 within the family of M17 aminopeptidases (M17-LAPs) which are characterized by having a highly conserved hexameric structure, and the CaLAP2 was grouped with other APs of the M1 family, which exist in monomeric or dimeric forms (Matsui et al 2006; Peer 2011; Drinkwater et al. 2019). On the contrary, intron size seems to be correlated with the rate of evolution and the regulation of genome size (Wendel et al. 2002). It has been suggested that plants with small genomes have smaller introns (Vinogradov 1999). The pepper genome has a size of about 3.5 Gb, being one of the largest ones in the Solanaceae family which is characterized to have largely of repetitive elements, estimated at 75–80% of the genome (Hulse-Kemp et al. 2018). In the case of *CaLAP* genes, it is remarkable that the genomic organization of *CaLAP* genes displays huge variations, particularly the *CaLAP2* with a very long intron expanding above 50 kb. This very long intron size is unusual in plants because large introns exceeding 1 kb are fewer in number (Wu et al. 2013). For example, in *Arabidopsis*, less than 1% of introns are longer than 1 kb (Chang et al. 2017).

During the ripening of pepper fruits and under an enriched environment of NO gas, we have described that a significant group of enzymes involved in the metabolism of ROS and RNS are modulated at different levels since this process has associated a nitro-oxidative stress (Corpas et al. 2018). The present data indicate that during ripening, the expression of both *CaLAP* genes is also differentially regulated. Thus, whereas *CaLAP1* decreased, *CaLAP2* experienced an increase during fruit ripening, and both genes were negatively modulated by a NO-enriched environment. Likewise, the total LAP activity during ripening showed an increasing activity, being significantly higher in ripe fruits. Contradictions between gene expression and the activity of the encoded proteins are not infrequent and suggest that post-translational modification could have a relevant functional regulation. The study of LAP activity has been studied in other fruits. In the non-climacteric grapefruits, the activity also increased along with ripening (Kang et al. 1999) and, more recently, similar behavior has been described in the climacteric durian fruit (Panpetch and Sirikantaramas 2021). Therefore, the obtained data on CaLAP activity from pepper fruits are in good agreement with those described in other fruits. This increase should be related to the drastic cellular changes that experimented on fruits during ripening, being the most relevant to the disassembling of chloroplasts and the formation of chromoplasts (Ling et al. 2021; Rödiger et al. 2021), which involves an intense proteolytic activity (Rodriguez-Concepcion et al. 2019; Ling et al. 2021).

In addition to the function of LAP in free amino acid regulation and protein turnover (Bartling and Weiler 1992; Kirmizi and Güleryüz, 2006), the enzyme participates in the response to wounding and pathogens (Pautot et al. 2001; Duprez et al. 2014; Fowler et al. 2009), and it has even been suggested that APs could be involved in the regulation of auxin transport (Murphy et al. 2000) and exhibit chaperone activity in tomato (Duprez et al. 2016; Scranton et al. 2012). Likewise, biochemical studies indicate that the LAP can exert its activity on dipeptides such as Cys–Gly and tripeptides in animal and plant systems (Cappiello et al. 2004; Chu et al. 2008; Gu and Walling 2000; Kumar et al. 2015). More recently, AP activity has been correlated with GSH recycling (Ito and Ohkama-Ohtsu 2023) during ripening (Panpetch and Sirikantaramas 2021). Furthermore, it has been shown that Cys–Gly promotes the formation of ROS such as H_2_O_2_ and superoxide anion in the presence of certain metal ions (Del Corso et al. 2002). In this context, we can hypothesize that the possible function of LAP activity, which increases during the ripening of pepper fruits, could be also associated with the metabolism of GSH, whose content decreases significantly during pepper ripening and thus contributing to the cellular redox state (González-Gordo et al. 2019). On the contrary, the fact that the pepper LAP activity in fruit is inhibited by GSH and l-Cys, two compounds that contribute to modulating the cellular redox state, could support this relationship between LAP activity and GSH.

**Fig. 8:**
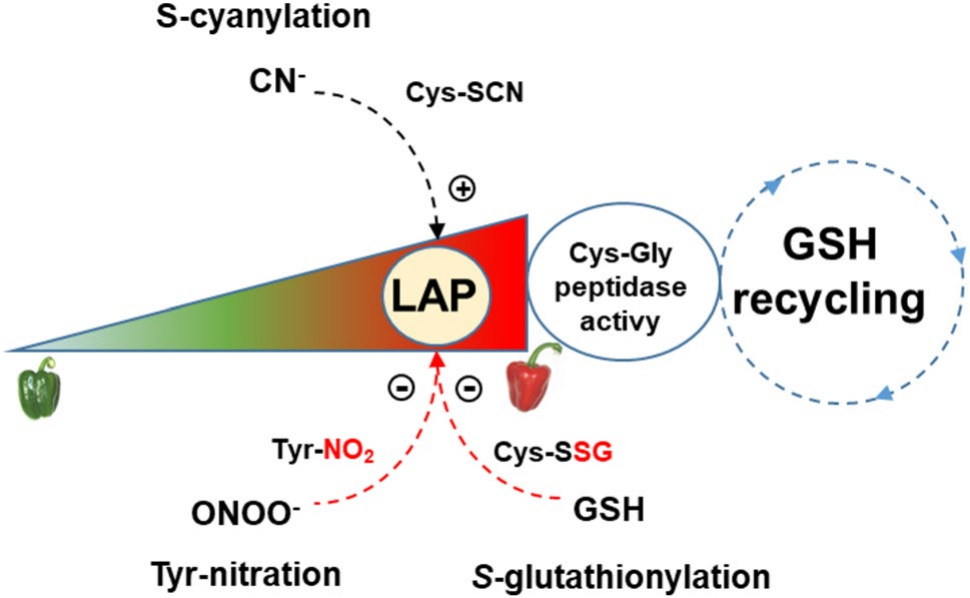
Working model of the possible post-translational modifications (nitration, *S*-glutathionylation, and *S*-cyanylation) that can regulate the CaLAP activity during the ripening of pepper fruits where the enzyme could participate in the GSH recycling process through its Cys–Gly peptidase activity

Another factor to be considered in this framework is that during pepper ripening there is an increase in proteins that can undergo a nitration process, including catalase (Chaki et al. 2015), NADPH-generating isocitrate dehydrogenase (Muñoz-Vargas et al. 2018), APX (González-Gordo et al. 2022), or the H_2_S-generating cytosolic l-Cys desulfhydrase (Muñoz-Vargas et al. 2023a). According to the obtained results in this work, LAP could be one of these nitrated proteins that are negatively affected in its activity. What is remarkable is the positive modulation of CaLAP activity by KCN. In this sense, it has been described in *Arabidopsis* that HCN can regulate cellular processes through thiol-based oxidative post-translational modifications (oxiPTMs) named *S*-cyanylation (García et al. 2019). This PTM can compete in the regulation of protein function with other oxiPTMs such as *S*-nitrosation, persulfidation, or *S*-glutathionylation, which are mediated by NO, H_2_S, and GSH, respectively (Niu et al. 2019; Corpas et al. 2022b). Based on the Cys–Gly peptidase activity of LAP described in *Arabidopsis* (Kumar et al. 2015), the pattern observed during the ripening of durian fruits (Panpetch and Sirikantaramas 2021), and the obtained data in the present work, a working model has been proposed (Fig. 8). Thus, one of the potential functions of the CaLAP could be its participation in the GSH recycling during the ripening of pepper fruits, and this could be modulated by several PTMs mediated by different compounds including ONOO^−^, GSH, and cyanide. Overall, it can be concluded that the LAP system could play an important role in the redox homeostasis of pepper fruit, a condition that gains relevance during ripening to maintain the fruit metabolism and preserve seeds to ensure the next generation.

### Supplementary Information

Below is the link to the electronic supplementary material.

Supplementary file1 (DOCX 532 KB)

## Data Availability

Sequence Read Archive (SRA) data are available at the following link: https://www.ncbi.nlm.nih.gov/sra/PRJNA668052 (accessed on May 28, 2020).
