# Supplementary material for: Characterization of leucine aminopeptidase (LAP) activity in sweet pepper fruits during ripening and its inhibition by nitration and reducing events
Source: Plant Cell Rep. 2024 Mar 11;43(4):92. doi: 10.1007/s00299-024-03179-x (PMC10927865; doi:10.1007/s00299-024-03179-x)
Supplement: Supplementary file 1 — Supplementary file1 (DOCX 532 KB) [file 299_2024_3179_MOESM1_ESM.docx]

**Supplementary Table S1.** **List of the 15 different LAP isozymes belonging to 8 different plant species used in this work for phylogenetic analysis,**

| **Organ/Plant species** | **LAP isozymes** | **Protein ID** | **Reference** |
| --- | --- | --- | --- |
| Fruit/Pepper (*Capsicum annuum)* | **CaLAP1**  **CaLAP2** | **XP_016540297.2**  **XP_016547301.1** | Present study |
| Leaf/*Arabidopsis thaliana* | AtLAP1  AtLAP2  AtLAP3 | NP_001189587.1  NP_194821.1  NP_001328632.1 | Polge et al., 2009 |
| Leaf/Rice (*Oryza sativa*) | OsLAP1  OsLAP2  OsLAP3 | Q2QSB9  Q6K669  Q84TA3 | Sasaki, 2005 |
| Leaf/Tomato (*Solanum lycopersicum*) | SlLAP1  SlLAP2 | AAC49456.1  AAC49457.1 | Gu et al., 1996 |
| Bud, flower, tuber, root and leaf/ Potato (*Solanum tuberosum*) | StLAP1  StLAP2 | XP_006350102.1  XP_015165363. | Herbers et al., 1994 |
| Root and leaf/  Tobacco (*Nicotiana*  *tabacum*) | NtLAP1 | XP_016443179.1 | Sierro et al., 2014 |
| Leaf/  Grape (*Vitis vinifera*) | VvLAP1 | XP_002276114.1 | Roach et al., 2018 |
| Leaf/  Pea (*Pisum sativum*) | PsLAP1  PsLAP2 | A0A9D4YLM9  A0A9D4WFU1 | Yang et al., 2022 |
| Fruit/Durian  (*Durio zibethinus*) | DzLAP1  DzLAP2 | MN879753 NW_019168159 | Panpetch P, Sirikantaramas, 2021 |
| Cassava (*Manihot esculenta*) | MeLAP1 | XP_021600337.1 | Alves-Pereira et al., 2022 |
| Durian (*Durio zibethinus*) | DzLAP1  DzLAP2 | XP_022750260.1  XP_022729747.1 | Teh et al., 2017 |
| Olive tree (*Olea europaea*) | OeLAP1 | CAA2965681.1 | Julca et al., 2010 |

**References**

Alves-Pereira A, Zucchi MI, Clement CR, Viana JP, Pinheiro JB, Veasey EA, de Souza AP (2022) Selective signatures and high genome-wide diversity in traditional Brazilian manioc (*Manihot esculenta* Crantz) varieties. Scientific Reports, 12(1).

Gu Y-Q, Chao WS, Walling L. (1996) Localization and post-translational processing of the wound-induced leucine aminopeptidase proteins of tomato. J Biol Chem. 271:25880–25887.

Herbers K, Prat S, Willmitzer L (1994) Functional analysis of a leucine aminopeptidase from *Solanum tuberosum* L. Planta. 194(2):230–240

Julca I, Marcet-Houben M, Cruz F, Gómez-Garrido J, Gaut BS, Díez CM, Gut IG, Alioto TS, Vargas P, Gabaldón T (2020) Genomic evidence for recurrent genetic admixture during the domestication of Mediterranean olive trees (*Olea europaea* L.). BMC biology, 18(1), 148.

Panpetch P, Sirikantaramas S (2021) Fruit ripening-associated leucylaminopeptidase with cysteinylglycine dipeptidase activity from durian suggests its involvement in glutathione recycling. BMC Plant Biol. 211:69.

Polge C, Jaquinod M, Holzer F, Bourguignon J, Walling L, Brouquisse R (2009) Evidence for the existence in *Arabidopsis thaliana* of the proteasome proteolytic pathway. Journal of Biological Chemistry, 284(51), 35412–35424.

Roach, M. J., Johnson, D. L., Bohlmann, J., van Vuuren, H. J., Jones, S. J., Pretorius, I. S., Schmidt, S. A., Borneman AR. (2018) Population sequencing reveals clonal diversity and ancestral inbreeding in the grapevine cultivar Chardonnay. PLOS Genetics, 1411.

Sasaki, T. 2005. The map-based sequence of the rice genome. Nature, 4367052), 793–800.

Sierro N, Battey JN, Ouadi S, Bakaher N, Bovet L, Willig A, Goepfert S, Peitsch MC, Ivanov NV (2014) The tobacco genome sequence and its comparison with those of tomato and potato. Nat Commun. 5:3833.

Teh BT, Lim K, Yong, CH, Ng CCY, Rao SR, Rajasegaran V, Lim WK, Ong CK, Chan K, Cheng VKY, Soh PS, Swarup S, Rozen SG, Nagarajan N, Tan P (2017) The draft genome of tropical fruit durian (*Durio zibethinus*). Nature genetics, 49(11), 1633–1641.

Yang T, Liu R, Luo Y, Hu S, Wang D, Wang C, Pandey MK, Ge S, Xu Q, Li N, Li G, Huang Y, Saxena RK, Ji Y, Li M, Yan X, He Y, Liu Y, Wang X, Xiang C, Varshney RK, Ding H, Gao S, Zong X (2022) Improved pea reference genome and pan-genome highlight genomic features and evolutionary characteristics. Nature Genetics, 5410), 1553–1563.

**Supplementary Figure S1.** Representative picture of the experimental strategy used in this study with the phenotype of sweet pepper (*Capsicum annuum* L.) fruits at different stages and treatments: immature green, breaking point 1 (BP1), breaking point 2 without nitric oxide (NO) treatment (BP2 – NO), breaking point 2 with NO treatment (BP2 + NO), and ripe red. Pepper fruits were subjected to a NO-enriched atmosphere (5 ppm) in a methacrylate box for one hour and were then stored at room temperature (RT) for 3 days. Reproduced with permission from González-Gordo et al. (2020).

**
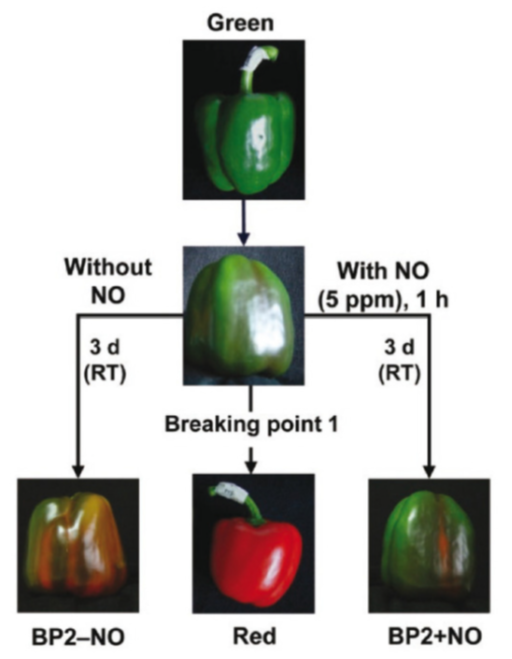
**

**Supplementary Figure S2.** Multiple alignment of LAP protein sequences from *Capsisum annuum* L. (CaLAP1), *Solanum lycopersicum* (SlLAP1 and SlLAP2), *Durio zibethinus* (DzLAP1), and *Olea europea* (OeLAP2). In the LAP proteins, there are eight highly conserved residues involved in substrate binding or catalytic function, corresponding in the CaLAP1 to K350, D357, K364, D377, D437, E439, R441, and L465 (labelled in red bold letters). Furthermore, five of these eight residues are conserved metal ion-coordinating residues (brown boxes). It is also indicated the Y318 residue which is a candidate for nitration. The asterisk (*) indicates positions that have fully conserved residue. The colon (:) denotes conservation between residues with strong similar properties. The dot (·) indicates conservation between residues with weakly similar properties.

**
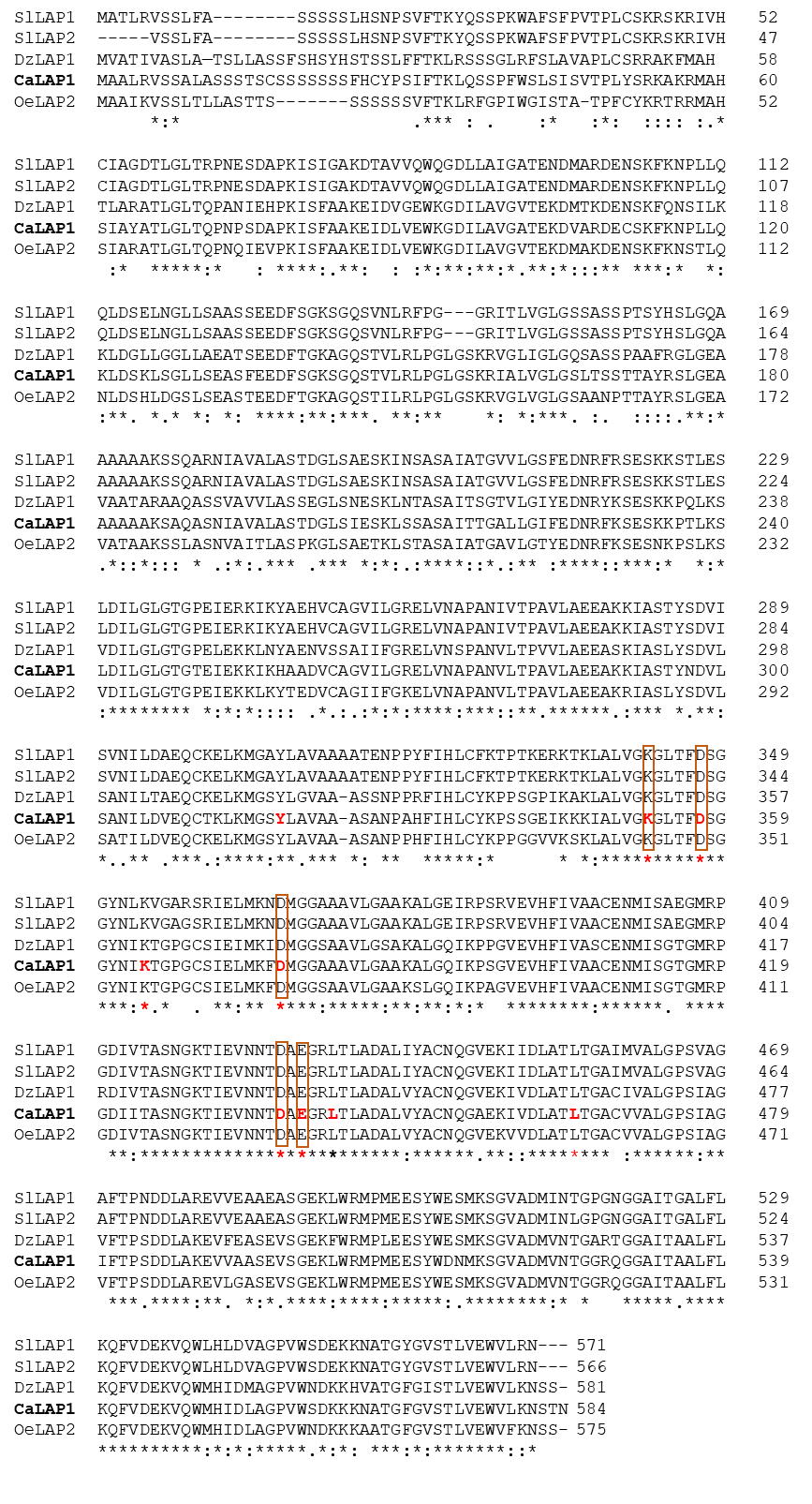
**
